# Food fraud threats in UK post-harvest seafood supply chains; an assessment of current vulnerabilities

**DOI:** 10.1038/s41538-024-00272-z

**Published:** 2024-05-27

**Authors:** Sophie Lawrence, Christopher Elliott, Wim Huisman, Moira Dean, Saskia van Ruth

**Affiliations:** 1 Institute for Global Food Security, School of Biological Sciences, 19 Chlorine Gardens, Queens University Belfast, Belfast, BT9 5DL Northern Ireland UK; 2 Faculty of Law, VU University Amsterdam, De Boelelaan 1105, Amsterdam, 1081 HV The Netherlands; 3 School of Agriculture and Food Science, University College Dublin, Dublin, 4 Ireland

**Keywords:** Agriculture, Ocean sciences, Interdisciplinary studies, Science, technology and society

## Abstract

Seafood fraud is commonly reported on food fraud databases and deceptive practices are highlighted by numerous studies, with impacts on the economy, health and marine conservation. Food fraud assessments are a widely accepted fraud mitigation and prevention activity undertaken to identify possible points of deception within a supply chain. This study aims to understand the food fraud vulnerability of post-harvest seafood supply chains in the UK and determine if there are differences according to commodity, supply chain node, business size and certification status. The SSAFE food fraud vulnerability assessment tool was used to assess 48 fraud factors relating to opportunities, motivations and controls. The analysis found seafood supply chains to have a medium vulnerability to food fraud, with the highest perceived vulnerability in technical opportunities. Certification status was a stronger determinant of vulnerability than any other factor, particularly in the level of controls, a factor that also indicated a higher perceived level of vulnerability in smaller companies and the food service industry. This paper also reviews historic food fraud trends in the sector to provide additional insights and the analysis indicates that certain areas of the supply chain, including uncertified prawn supply chains, salmon supply chains and food service companies, may be at higher risk of food fraud. This study conducts an in-depth examination of food fraud vulnerability relating to the UK and for seafood supply chains and contributes to a growing body of literature identifying areas of vulnerability and resilience to food related criminality within the global food system.

## Introduction

Food fraud has a well-documented history^1^ and provides profitable opportunity for those who choose to deceive. Following several high-profile food fraud scandals, food-related criminality is widely acknowledged as an issue of concern for the food industry, consumers, and regulators, with implications for the economy, public health and consumer confidence^2–4^.

Seafood, included as an enforcement priority in the UK and Scotland^5,6^ is a group of commodities with an established history of food fraud^7^ and evidence of global seafood mislabelling is described as ‘ubiquitous’^8^. With one of the most complex and diverse sets of supply chains in the food industry and facing increased demand and resource scarcity, the seafood sector faces a considerable challenge in ensuring the integrity of its products^9^. EU exit, the COVID-19 pandemic, the war in Ukraine and inflation have placed further pressure on supply chains and risk factors for food fraud are likely to be elevated^10–12^. Seafood fraud is revealed at every node in the supply chain through a wide variety of methods^7,13^. It has negative consequences for sustainable and ethical marine management^14,15^ and also poses a risk to public health due to the presence of toxins, contaminants, allergens or zoonotic parasites^16,17^.

Food fraud mitigation and prevention are supported by international and intergovernmental organisations and networks^18–22^, domestic regulatory authorities^5,23^ and are the focus of a growing body of research^24–28^. However, it is the food industry that must ensure that adequate systems are in place to ensure the integrity of their supply chain^29^ and legislation requires food businesses to ensure the safety and quality of their products that are not labelled in a false or misleading way. In the UK, this is governed predominantly by the Food Safety Act (1990), amongst other legislation and is enforced by the Food Standards Authority in England and Wales and in Scotland, Food Standards Scotland.

Food fraud vulnerability exists at weak spots in a supply chain where motivation or opportunity are raised, or controls are lacking and is dependent on multiple factors; for example, the inherent vulnerability of certain commodity chains to food fraud and the availability of supply, as well as company and sector characteristics that could increase the likelihood of fraudulent activity, such as the economic landscape, business culture and local food safety legislation and its enforcement^26,30^.

Therefore, a situational, structured approach that considers the wider environment and context of food chain actors, their markets, processes and products, along with regional or sectorial countermeasure or control systems, is fundamental to predict where issues may arise^31^. Few other business environments incorporate such a diverse group of actors and power relations^32,33^^,^ whose motivation to engage in criminality can be vastly different. Businesses are influenced by economic, social and cultural influences that interact to propel offenders into criminality – fraud may be driven simply by financial greed or as a last-ditch response to market and supply chain pressures^34^.

Food fraud vulnerability assessments take a systematic view of these factors within an individual business environment or supply chain to indicate where there is resilience or identify and prioritise food fraud vulnerability, defined by the Global Food Safety Initiative as ‘the susceptibility or exposure to a food fraud risk, which is regarded as a gap or deficiency that could place consumer health at risk if not addressed’^35^. There are several tools available for FFVA^36^, including the SSAFE FFVA tool^37^, the Food Fraud Advisor’s Vulnerability Assessment Tool^38^ and EMAlert—Economically Motivated Adulteration Vulnerability Assessment Tool^39^.

Developed for company level assessment, the use of their application to country and sector level analysis is recognised in the literature^7,40^ and vulnerability assessments have been used and adapted by the academic community and others to compare fraud drivers, enablers and control measures across sectors, actor groups and geographies. Assessments have been conducted for spices^41^, a comparison of different supply chains^42^, Dutch milk^43^, olive oil^44^, Chinese milk^45^, Chinese rice^46^ and the food service^47^ and organic sectors^48^. The results are summarised in a a recent paper^40^. There is agreement that vulnerability assessments of the seafood sector could help uncover and mitigate or prevent fraudulent opportunities within seafood supply chains^7^. Food fraud assessments have not yet been conducted in-depth for seafood, in the UK or elsewhere, so this research aims to address this gap.

Comparing associations between food fraud vulnerability and historic food fraud incidents can provide further insights^45,49,50^. For example, areas of a supply chain or products that indicate both food fraud vulnerability and high levels of historic fraud should demand closer scrutiny and present an opportunity to implement countermeasures, whereas areas that indicate vulnerability, but not historic levels of reported fraud may represent an emerging threat. This study therefore considers the food fraud vulnerabilities in cod, prawn, and salmon supply chains according to business characteristics, and how these vulnerabilities relate to historical criminal activity in these sectors.

## Results

Fraud vulnerabilities were collated for the 32 companies and the frequencies of high, medium and low vulnerability responses calculated. Vulnerability profiles according to opportunities, motivations and controls were analysed across the whole dataset. Groupings within the data are explored via multiple correspondence analysis (MCA) and differences in vulnerability by commodity, supply chain node, business size and certification status are assessed.

### Overall food fraud vulnerability

Across the whole dataset, low vulnerability was the most common response, accounting for 45% of all responses. 28% were rated medium vulnerability and 27% as highly vulnerable.

**Fig. 1:**
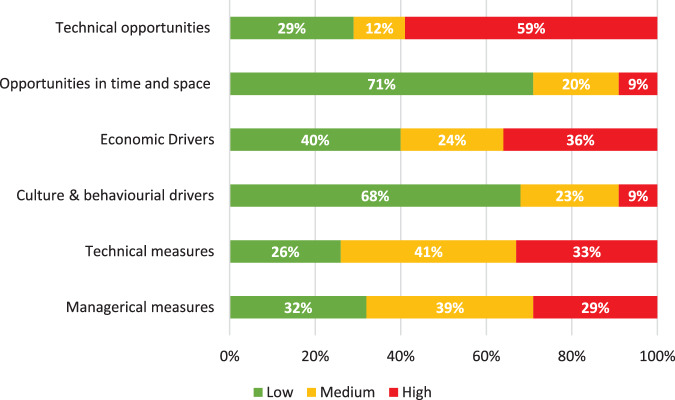
The percentage of low, medium and high vulnerability scores by fraud factor category, for all participants (*n* = 32). Low vulnerability scores are green, medium vulnerability are orange and high vulnerability scores are red. This figure shows the distribution of vulnerability scores by opportunities related fraud factors (technical opportunity, opportunity in time and space), motivations related fraud factors (economic drivers, cultural and behavioural drivers) and control measures (technical measures, managerial measures).

Figure 1 shows the proportion of vulnerability scores broken down into opportunities related fraud factors (technical opportunity, opportunity in time and space), motivations related fraud factors (economic drivers, cultural and behavioural drivers) and control measures (technical measures, managerial measures). The full breakdown of responses by individual fraud factor is available in Supplementary Information A, Table 1.

**Table 1:** Characteristics of the UK-based survey respondents

Supply chain node	Company size	Location	Commodity	Third party accreditation (own company or for suppliers)
Processor 1	Small	South East	Salmon	No
Processor 2	Small	South East	Salmon	No
Processor 3	Small	Yorks/ Humber	Prawns	Yes
Processor 4	Medium	Yorks/ Humber	Cod	Yes
Processor 5	Medium	Scotland	Salmon	Yes
Processor 6	Medium	South West	Cod	Yes
Processor 7	Large	Scotland	Salmon	Yes
Processor 8	Large	Yorks/ Humber	Prawns	Yes
Processor 9	Large	Northern Ireland	Prawns	Yes
Processor 10	Large	National	Salmon	Yes
Wholesaler 1	Small	London	Cod	Yes
Wholesaler 2	Small	Yorks/ HumbeSouth West	Cod	Yes
Wholesaler 3	Small	South East	Cod	No
Wholesaler 4	Small	Wales	Prawns	Yes
Wholesaler 5	Small	Yorks/Humber	Salmon	No
Wholesaler 6	Medium	Yorks/Humber	Salmon	No
Wholesaler 7	Medium	North West	Cod	Yes
Wholesaler 8	Large	National	Cod	No
Retailer 1	Small	South East	Cod	No
Retailer 2	Small	East of England	Cod	No
Retailer 3	Small	South West	Cod	No
Retailer 4	Small	South East	Prawns	No
Retailer 5	Small	South East	Salmon	No
Retailer 6	Large	National	Prawns	Yes
Retailer 7	Large	National	Prawns	Yes
Retailer 8	Large	National	Prawns	Yes
Food service 1	Small	Northern Ireland	Prawns	No
Food service 2	Small	East of England	Prawns	No
Food service 3	Small	North West	Cod	No
Food service 4	Large	National	Cod	No
Food service 5	Large	National	Salmon	Yes
Food service 6	Small	Scotland	Salmon	No

The highest frequency of high vulnerability responses was observed in technical opportunities, whilst opportunities in time and space had the lowest. Motivations had the overall lowest number of high vulnerability responses.

### Opportunities (Low 48%, medium 15%, high, 37%)

Within technical opportunities, the complexity of adulteration of raw materials within the fish supply chain was rated lower vulnerability than other technical related fraud factors since respondents generally bought their raw product in whole fish form or fillets. The available technology and knowledge to adulterate or substitute raw and final materials was perceived as highly vulnerable, as adding or exchanging one morphologically similar species for another requires little specialist knowledge or technology.

Notably, the perception of vulnerability for raw products was much higher (low 16%, medium 9%, high, 75%) in contrast to the availability, technology and knowledge to adulterate final products (low 32%, medium 23%, high 45%), even in processing, an area of the supply chain that offers a broad opportunity for adulteration. The possibility of adulteration in final product form assumes the possibility of fraud occurring onsite. Other studies on food fraud vulnerability have observed that responses may be answered more cautiously when the questions concern the own company^49^^,50^ and the possibility of internal threats more difficult to comprehend than external ones. In terms of detection, both raw and final products scored high vulnerability (66% and 71% high vulnerability scores, respectively), reflecting the complexity of fish fraud identification^51^^,52^.

Fraud opportunities in time and place were perceived to be less vulnerable than technical opportunities. Where applicable, production lines were operated with a high level of control and minimal opportunity for unauthorised interference (low 71%, medium 19%, high 10%). Given the complexity of seafood supply chains, respondents felt they had good insight into suppliers and customers and were generally categorised by longstanding, trusted relationships (low 88%, medium 6%, high 6%). It was not perceived that fraudulent reports of raw or final products for cod, salmon or prawns were commonly reported, with over 60% of respondents rating low vulnerability for these fraud factors, a perception that stands in contrast to the levels of fraud evidenced in the literature and the media^7,8,13,52^.

### Motivations (Low 57%, medium 23%, high 18%)

Attributed value according to seafood production methods or origin scored the highest vulnerability from all the 48 fraud factors (low 19%, medium 3%, high 78%), as consumers show preference and willingness for credence attributes such as locally caught, wild, organic or sustainably sourced seafood^53^.

Price differentials due to regulatory differences between countries also generated high vulnerability responses (low 13%, medium 33%, high 54%). Vulnerability levels due to price instabilities and shortages in supply and pricing varied, depending on the product and geographical origin. Covid-19, EU exit and the war in Ukraine have caused market disruption and a tightening in supply of certain seafood products^10–12^.

Competition in the industry was also rated as high vulnerability, with 72% of respondents indicating that their sector of the supply chain was highly competitive. Despite these challenges, companies currently considered themselves (84%) and their suppliers (65%) profitable. Companies did not generally perceive that they imposed financial strains on suppliers, with 66% of responses as low vulnerability and except for one respondent, suppliers were not dependent on the company for their financial survival.

Cultural and behavioural drivers were generally rated as low vulnerability. Both own company and supplier business strategy and ethical business culture scored over 80% of answers as low vulnerability. The scores companies attributed to the ethical business culture across their industry (i.e., their company and their competitors) were much lower (low 47%, medium 34%, high 19%). This ordering is reflected in other studies on food fraud vulnerability where companies have rated their own company as lowest vulnerability, followed by their suppliers, then industry^43,48^. Questioning ethical business strategy is sensitive, firm-specific and potentially open to social desirability bias^54^. Relative analysis between commodity, supply chain node, business size and certification is presented in the section The Influence of Company Characteristics on Food Fraud Vulnerability to mitigate this as far as possible.

Historic levels of criminality in the sectors (processing, wholesale, retail or food service) were perceived as relatively low. High vulnerability scores for criminal offences of own company were 3% and for suppliers were 7%. Suppliers had been a victim of food fraud in 8% of responses.

### Controls (Low 28%, medium 40%, high 30%)

In general, technical controls were rated as medium vulnerability. 38% of companies had a comprehensive monitoring control system, including a systematic evidence-based sampling plan with specific fraud screening methods and systematic record keeping, but 34% of companies had no methods for fraud detection in place. A lower vulnerability was attributed to the control of mass balance flows, with 54% of responses reporting established and comprehensive mass balance data. This was even higher for suppliers at 74%, mostly due to the requirements of third-party certification for mass balance flows^55^. Fraud contingency planning was more vulnerable, with only 33% of responses with a contingency plan that included fraud incidents. 47% of responses had no contingency plans for fraud or safety issues.

Managerial measures had varying levels of vulnerability. Ethical codes of conduct were implemented by 78% of companies and 56% of these were embedded across the company. A whistleblowing system was in place for 60% of companies, although only 47% provided an independent reporting line and assured anonymity. Where there is no independent reporting for qualified disclosures, employees may be required to report to their immediate supervisors, which may act as a deterrent to whistleblowing and put them at risk. Integrity screening of employees was less common with 65% of companies having no integrity screening in place. It was perceived that the national food fraud policy was relatively comprehensive (low 59%, medium 24%, high 17%) but that local fraud prevention laws were less stringently enforced. 59% of companies rated this as medium vulnerability with a low frequency of inspections and low-level fines/sanctions with little financial impact.

Also notable was the perceived lack of guidance for fraud prevention and control across various sectors of the supply chain. 33% of companies were unaware of any guidance for fraud mitigation and an additional 30% did not feel that there were adequate guidelines with specific training and examples of best practice. This was particularly prevalent for smaller companies and food service companies. Various free training resources exist that are accessible and free for smaller businesses^23,56,57^, but conducting this assessment provided valuable insights - many smaller companies were not aware of them. Increasing awareness through outreach programs, social media, or industry bodies, providing tangible examples of best practices in the food industry and perhaps simplified tools to provide entry-level access to food fraud resources could help these companies emulate robust fraud mitigation practices.

### Food fraud vulnerability data—exploring associations

**Fig. 2:**
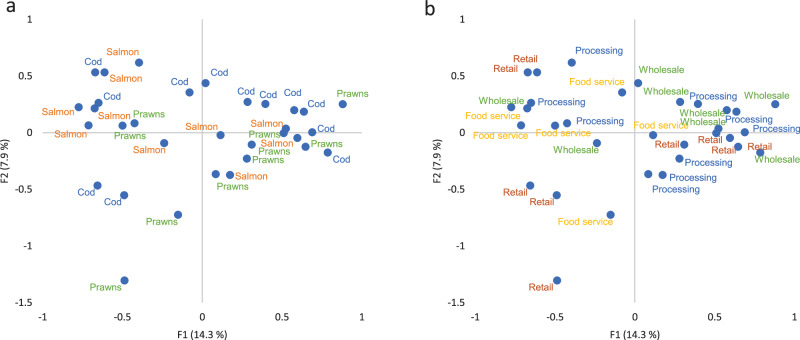
Scores plots for the first two dimensions of multiple correspondence analysis for the FFVA for 32 seafood companies by commodity and supply chain node. This figure illustrates the associations discovered through multiple correspondence analysis according to commodity (**a**) and supply chain node (**b**).

**Fig. 3:**
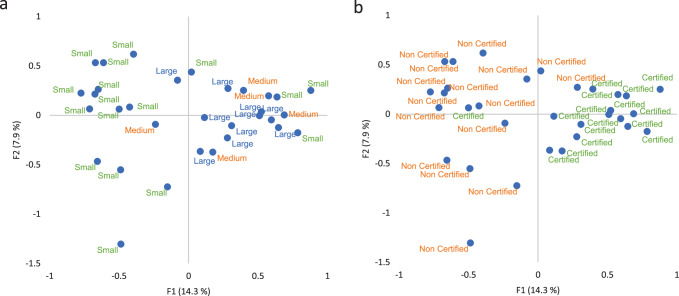
Scores plots for the first two dimensions of multiple correspondence analysis for the FFVA for 32 seafood companies by business size and certification status. This figure illustrates the associations discovered through multiple correspondence analysis according to business size (**a**) and certification status (**b**).

A multiple correspondence analysis was conducted on the dataset to provide an initial representation of groupings within the data. The first two dimensions by various categories are presented in Fig. 2 and Fig. 3. Each dimension captures patterns and associations between categories within variables, with the first dimension explaining the most variance. The first dimension (F1) explains 14.3% of the variance and the second dimension (F2) 7.9%. The values on the X and Y axes represent the positions of categories or categories of variables in the reduced-dimensional space created by the analysis.

In MCA diagrams 2a and 2b, little association is observed by commodity and supply chain node. Further analysis of these categories is undertaken in the following section to see if there are commonalities or differences between individual fraud factors and fraud factor categories.

Figure 3 explores associations by business size and certification status and more observable patterns are present in this analysis. Diagram 3a demonstrates some clustering by business size, separated mainly by the first dimension, with small companies on the left-hand side and medium and large companies on the right-hand side, although some small companies also occupy the right-hand side. Diagram 3b shows two distinct groups, also separated by the first dimension that groups non-certified companies on the left-hand side and certified companies on the right-hand side.

**Fig. 4:**
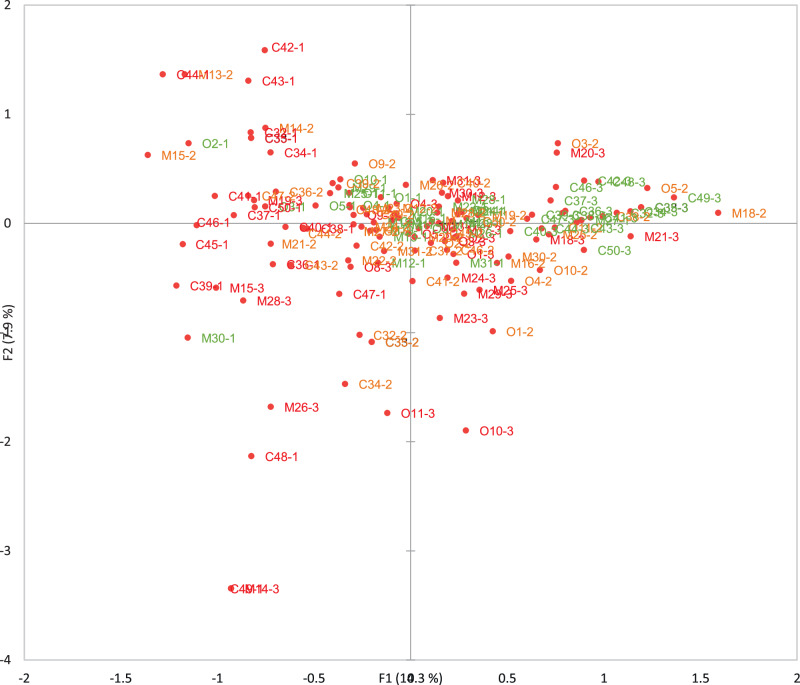
Loadings plot of multiple correspondence analysis for the FFVA for 32 seafood companies. Within the plot, O represents opportunities, M represents motivations and C represents control measures. For opportunities and motivations (Q1-32), vulnerability levels are coded as follows: 1 = low vulnerability, 2 = medium vulnerability and 3 = high vulnerability. Conversely, for controls (Q32-50), these scores are reversed. The vulnerability levels are colour-coded for clarity; green indicates lower vulnerability, orange represents medium vulnerability and red signifies high vulnerability.

According to the loadings plot in Fig. 4, these groupings are largely due to control-related fraud factors, with higher levels of vulnerability on the left-hand side and lower levels on the right-hand side, indicating that larger companies and certified companies may have more comprehensive control systems in place.

### The influence of company characteristics on food fraud vulnerability—a statistical evaluation of commodity, supply chain node, business size and certification status

Mann-Whitney U and Kruskal Wallis tests were run to compare significant differences (*p* < 0.05) by all factors (commodity, supply chain node, business size and certification status). The difference in perceived fraud vulnerability was determined separately for commodity, supply chain node, business size and certification status for each of the fraud factors. The *P* values and mean rank of fraud factors are presented in Table 2 and Table 3. For opportunities and motivations, a higher rank indicates higher vulnerability, whilst for controls, a higher rank indicates lower vulnerability.

**Table 2:** Significance of differences (*p* < 0.05) and mean ranks of fraud vulnerability scores for individual fraud factors based on commodity and supply chain node

		Differences due to product				Differences due to supply chain node				
No.	Fraud factor	Mean rank			Asymp. Sig	Mean rank				Asymp. Sig
		Cod	Prawns	Salmon		Processing	Wholesale	Retail	Food service	
1	Complexity of adulteration of raw materials	12	25	13	<0.001*	15	15	19	18	0.630
2	Availability technology and knowledge to adulterate raw materials	18	19	13	0.093	14	21	14	18	0.180
3	Fraud detectability in raw materials	14	21	15	0.110	16	16	15	20	0.682
4	Technology and knowledge to adulterate final products	18	16	16	0.816	12	23	15	17	0.089
5	Fraud detectability in final products	16	17	16	0.931	13	18	16	22	0.195
8	Access to production lines/processing activities	16	15	19	0.423	n/a	n/a	n/a	n/a	n/a
9	Transparency in the chain network	16	18	16	0.684	16	15	18	17	0.548
10	Historical evidence of fraud in raw materials	16	20	14	0.175	17	17	19	13	0.614
11	Historical evidence of fraud in final products	16	18	15	0.620	16	17	19	13	0.544
12	Supply and pricing raw materials	19	18	12	0.146	17	17	16	16	0.991
13	Valuable components or attributes	18	15	17	0.685	18	16	14	17	0.618
14	Economic health own company	15	17	17	0.667	17	18	16	14	0.655
19	Financial strains supplier	16	18	15	0.582	18	16	16	15	0.841
20	Economic health supplier	15	19	16	0.546	17	14	20	16	0.562
26	Economic health sector	14	18	18	0.558	13	17	16	22	0.283
30	Level of competition branch of industry	16	17	17	0.873	17	19	14	16	0.582
31	Price asymmetries	16	18	15	0.814	15	18	17	16	0.956
15	Organisational strategy own company	15	17	17	0.631	18	17	17	15	0.804
16	Ethical business culture own company	16	16	18	0.691	16	16	16	19	0.722
17	Criminal offences own company	16	16	18	0.333	16	18	16	17	0.228
18	Corruption level country own company	15	17	17	0.631	20	13	13	19	0.914
21	Organisational strategy supplier	14	18	18	0.373	20	14	16	17	0.155
22	Ethical business culture supplier	14	20	16	0.254	17	13	19	17	0.391
23	Criminal offences supplier	16	16	17	0.973	19	11	17	19	0.593
24	Victimisation of supplier	13	19	19	0.110	18	15	18	14	0.208
25	Corruption level country supplier	14	24	11	0.002*	13	17	16	22	0.665
27	Criminal offences customer	15	17	17	0.709	14	13	21	21	0.041*
28	Ethical business culture branch of industry	16	17	16	0.971	18	17	17	14	0.851
29	Historical evidence branch of industry	17	17	16	0.916	14	18	18	16	0.743
32	Fraud monitoring system raw materials	17	20	13	0.281	14	18	18	16	0.464
33	Verification of fraud monitoring system raw materials	17	18	14	0.530	18	19	18	10	0.297
34	Fraud monitoring system final products	17	20	12	0.178	18	18	19	9	0.165
35	Verification of fraud monitoring system final products	17	18	14	0.530	18	19	18	10	0.276
36	Information system own company	18	19	13	0.251	19	22	15	7	0.004*
37	Tracking and tracing system own company	19	18	13	0.229	19	21	15	9	0.016*
42	Fraud monitoring system supplier	21	16	12	0.017*	15	21	16	13	0.209
43	Information system supplier	17	17	15	0.866	18	22	13	12	0.031*
44	Tracking and tracing system supplier	17	16	16	0.907	18	21	14	12	0.069
50	Contingency plan	16	19	15	0.515	19	18	15	13	0.315
38	Integrity screening own employees	18	18	13	0.276	15	19	17	16	0.880
39	Ethical code of conduct own company	18	17	15	0.795	19	18	14	15	0.439
40	Whistle blowing own company	14	20	17	0.282	20	16	15	14	0.338
41	Contractual requirements supplier	18	18	13	0.366	18	19	15	13	0.559
45	Social control chain network	15	18	17	0.741	18	19	15	12	0.405
46	Fraud control industry	16	17	16	0.949	20	22	12	11	0.03*
47	National food policy	17	17	15	0.832	16	21	15	14	0.326
48	Law enforcement local chain	19	13	18	0.184	18	23	13	12	0.006*
49	Law enforcement chain network	20	15	15	0.093	16	22	13	15	0.020*

For opportunities and motivations, a higher rank indicates higher vulnerability, whilst for controls, a higher rank indicates lower vulnerability. For comparison by supply chain node, questions 8 and 27 were omitted as were not relevant for all supply chain nodes.

*indicates fraud factors with statistically significant differences (*p* < 0.05).

**Table 3:** Significance of differences (*p* < 0.05) and mean ranks of fraud vulnerability scores for individual fraud factors based on business size and certification status

		Differences due to size			Differences due to certification status		
No.	Fraud factor	Mean rank		Asymp. Sig	Certification status		Asymp. Sig
		Small	Large		Non-Certified	Certified	
1	Complexity of adulteration of raw materials	13	16	0.232	15	18	0.215
2	Availability technology and knowledge to adulterate raw materials	13	16	0.26	14	19	0.041*
3	Fraud detectability in raw materials	12	17	0.064	14	19	0.071
4	Technology and knowledge to adulterate final products	15	13	0.505	15	18	0.466
5	Fraud detectability in final products	14	15	0.534	16	17	0.563
8	Access to production lines/processing activities	15	12	0.305	20	14	0.051
9	Transparency in the chain network	14	15	0.569	16	17	0.324
10	Historical evidence of fraud in raw materials	14	14	1	14	19	0.084
11	Historical evidence of fraud in final products	15	13	0.411	15	18	0.378
12	Supply and pricing raw materials	14	15	0.743	15	18	0.439
13	Valuable components or attributes	13	16	0.267	16	17	0.599
14	Economic health own company	16	12	0.063	18	15	0.313
19	Financial strains supplier	14	15	0.774	14	19	0.119
20	Economic health supplier	13	16	0.256	17	16	0.606
26	Economic health sector	16	11	0.09	20	14	0.04*
30	Level of competition branch of industry	12	17	0.07	14	19	0.085
31	Price asymmetries	15	13	0.535	17	16	0.916
15	Organisational strategy own company	15	12	0.064	18	15	0.127
16	Ethical business culture own company	14	15	0.192	16	17	0.356
17	Criminal offences own company	14	15	0.192	16	17	0.348
18	Corruption level country own company	13	16	0.17	14	19	0.025*
21	Organisational strategy supplier	15	13	0.377	18	16	0.4
22	Ethical business culture supplier	14	13	0.782	17	16	0.814
23	Criminal offences supplier	15	13	0.632	17	16	0.763
24	Victimisation of supplier	13	15	0.535	17	17	1
25	Corruption level country supplier	12	18	0.036*	12	21	0.002*
27	Criminal offences customer	15	12	0.248	19	14	0.027*
28	Ethical business culture branch of industry	17	10	0.016*	20	14	0.065
29	Historical evidence branch of industry	14	14	0.909	16	17	0.54
32	Fraud monitoring system raw materials	12	18	0.054	9	23	<0.001*
33	Verification of fraud monitoring system raw materials	12	18	0.06	10	22	<0.001*
34	Fraud monitoring system final products	12	17	0.087	10	22	<0.001*
35	Verification of fraud monitoring system final products	12	18	0.06	10	22	<0.001*
36	Information system own company	12	17	0.075	12	21	0.02*
37	Tracking and tracing system own company	13	16	0.22	13	20	0.03*
42	Fraud monitoring system supplier	13	16	0.187	14	19	0.106
43	Information system supplier	12	18	0.04*	12	21	0.002*
44	Tracking and tracing system supplier	13	16	0.189	13	20	0.027*
50	Contingency plan	11	19	0.006*	11	22	<0.001*
38	Integrity screening own employees	11	20	<0.001*	12	21	<0.001*
39	Ethical code of conduct own company	11	20	0.002*	11	21	<0.001*
40	Whistle blowing own company	10	22	<0.001*	11	21	<0.001*
41	Contractual requirements supplier	11	20	0.001*	9	23	<0.001*
45	Social control chain network	15	13	0.456	16	17	0.758
46	Fraud control industry	12	18	0.055	11	21	<0.001*
47	National food policy	13	17	0.184	14	19	0.06
48	Law enforcement local chain	13	16	0.348	13	19	0.026*
49	Law enforcement chain network	15	13	0.416	14	19	0.015*

For opportunities and motivations, a higher rank indicates higher vulnerability, whilst for controls, a higher rank indicates lower vulnerability.

*indicates fraud factors with statistically significant differences (*p* < 0.05).

Cod, prawn and salmon food supply chains were compared to examine the influence by commodity and significant differences were identified for only 3 out of the 48 fraud factors. These included the complexity of adulteration of raw materials (Q1), country corruption level of suppliers (Q25) and the fraud monitoring system of suppliers (Q42). Prawns had a higher perceived vulnerability for Q1, due to the variety of methods by which they can be adulterated, including undeclared added ingredients for bulking and plumping, such as water-binding agents. There was a notable difference in the countries ranking on the TI corruption perception index (used as an indicator for the prevalence of financial and economic crime) where suppliers are active (Q25), depending on the commodity. Almost all the prawn supply chains in the responses were warm water prawns, farmed in Vietnam, China and India, with higher corruption levels^58^ than salmon and cod, generally caught or farmed in Europe. Salmon had a higher perceived vulnerability than the other two supply chains for the fraud monitoring system of their suppliers.

For supply chain node, differences were identified between processing, wholesale and distribution, retail and food service for five fraud factors, all related to the level of controls. Differences in technical controls included information system of own company (Q36), the tracking and tracing system of own company (Q37) and information system of supplier (Q43) with processing and wholesale having the most comprehensive systems, followed by retail and food service with the highest level of perceived vulnerability for these factors. Differences for managerial controls applied to industry guidance for fraud prevention and control (Q46) and local (Q48) and international (Q49) fraud related enforcement.

Retailers and food service were less aware of industry guidance for fraud prevention and control, with both supply chain nodes reporting high vulnerability for this fraud factor. There is a wealth of industry guidance available, but this response suggests a need for a more tailored approach to meet the needs of business owners less familiar with food fraud resources. Simplified assessments and information campaigns customised to industry could aid accessibility for all sizes and types of business.

Also notable in this section was a difference in knowledge of the fraud related systems of suppliers; the fraud monitoring system of suppliers (Q32), and traceability systems of suppliers (Q44)—only 38% of these questions were answered by food service companies, compared to 58% of retailers, 75% of wholesalers and 83% of processors. Where supplier controls and traceability are unknown, buyers have effectively lost control of their supply chain, providing ample opportunity for fraud to occur. Lack of knowledge in this regard indicates serious vulnerability and undermines the low vulnerability rating provided by those supply chain nodes for fraud factor 9 (transparency in the chain network). Increasing regulatory requirements for traceability systems that track the movement of seafood products from the point of production to point of sale could help close this gap in knowledge. Increasing consumer awareness of food fraud may help drive demand for sea to plate traceability at the point of sale.

Small and large-scale companies were compared and perceived fraud vulnerability differed according to business size for eight fraud factors. There was no statistically significant difference for technical opportunities, opportunities in time and space or economic drivers. There were two fraud factors with a significant difference in cultural and behavioural drivers; ethical business culture across branch of industry (Q28) where smaller companies perceived their sector of the food supply chain to have a lower ethical business culture than larger suppliers and the corruption levels of country suppliers (Q25), where larger companies had more exposure to suppliers in countries with higher corruption levels. Prawns, which were mostly imported, had a higher representation among larger companies in the dataset, accounting for 29% of the larger companies, which is likely to account for the difference.

A greater difference was observed by business size in terms of controls, mainly in managerial controls for the integrity screening of own employees (Q38), ethical code of conduct own company (Q39), whistleblowing own company (Q40) and contractual requirements supplier (Q41). Information system of suppliers (Q43) and fraud contingency plan (Q50) were the technical control related fraud factors with statistical difference. As noted in previous research, both comprehensive technical fraud monitoring systems and managerial measures such as integrity screening, code of conduct and robust whistleblowing practices are often more commonly observed in larger companies who have the money and resources to implement them^41,44^. However, as fraudsters are non-discriminatory regarding to size, all scales of company are vulnerable to deception, either from internal or external threats and increased awareness and support for smaller businesses could help improve both individual businesses and consequently the overall resilience of the supply chain to food fraud. Industry standards bodies are increasingly supporting this strategy, for example, GFSI’s Global Markets and BRCGS’s START! programs that aim to help smaller businesses achieve certification.

Certification status had the most substantial impact on statistical differences in fraud vulnerability between companies. Industry standards such as BRCGS and GFSI require updated food fraud vulnerability assessments and associated food fraud mitigation/prevention plans that are implemented. Sustainability certifications such as Marine Stewardship Council (MSC) or Aquaculture Stewardship Council (ASC) reduce information asymmetry along the supply chain and provide an additional layer of controls^59^, so this effect is not unexpected. However, it has not previously been evidenced in the literature. Twenty-one fraud factors showed a statistical difference. In opportunities, this included fraud factor 3 (the availability and knowledge to adulterate raw materials), with certified companies perceiving a higher vulnerability than non-certified ones. Non-certified companies perceived the economic health of the sector they belong to be lower than certified companies (Q26). In cultural and behavioural drivers, corruption level own company (Q18) and corruption level country of supplier (Q25) had a higher perceived vulnerability in certified companies due to a more internationally based company or supply chain. For criminal offences, customer (Q27) non-certified companies had a higher perceived level of vulnerability.

The greatest difference was in the level of controls, with 86% of fraud factors showing significant differences and, in all cases, certified companies with more robust controls than non-certified. The data showed statistical differences in every technical-related control measure, except for the fraud monitoring system of suppliers. For the supplier-related questions (Q42–44), the level of knowledge of supplier control systems was also notable. Non-certified companies were only able to answer 50% of responses on fraud factors in comparison to 91% of responses for certified companies.

For managerial controls, all own company fraud factors showed statistical difference (Q38–40) as well as contractual requirements supplier (Q41) and food fraud guidance (Q46), with uncertified companies less confident about fraud related resources than certified companies.

It should be noted, however, that this is just a broad exploration of the effect of certification on food fraud vulnerability factors. The analysis does not discriminate between standards, sustainability or ethical certification and the different protections that each certification category affords. Certified products may carry an associated price premium^60^ and/or increase access to certain markets^61^, so certification brings its own opportunity for food fraud. As the number and use of certification bodies increase^62^, careful monitoring by regulators will be required to sustain legitimacy and ensure practice reflects policy.

### Combining food fraud vulnerability findings with publicly available historic food fraud data

Findings from the food fraud vulnerability assessments were combined with publicly available data on food fraud between 31/01/2020 and 31/12/2020 to provide additional insights on fraudulent activity in the sector.

There were 240 incidences of fraud recorded on the databases; 193 for prawns, 26 for cod and 21 for salmon. For prawns, 80% of these records related to the presence of illegal or unauthorised veterinary residues, mostly in imported farmed seafood from Asia. Most of the respondents sourced prawns from Asian farms. However, over 70% of SSAFE vulnerability assessment respondents were certified, or products were certified and prawn supply chains had more comprehensive control measures than cod and salmon supply chains, so these risks may be mitigated. Uncertified prawn supply chains were found in smaller retailers and food service companies, with minimal control measures in place, so this could represent an area of vulnerability.

Historic fraud incidences in cod and salmon included the misrepresentation of weight, species and fishery substitution and adulteration. Salmon supply chains reported higher vulnerability regarding the fraud monitoring systems of their suppliers. An absence of control measures, along with fraud opportunity or motivation, may indicate an area that requires closer scrutiny. Inflationary pressures can add motive to supply chains where there is opportunity and enhancing control measures should be regarded as a high priority.

By supply chain node, historic fraud incidence data showed the highest prevalence of fraud in processing (39% of reports) and food service (42% of reports). The greatest differences within the responses of the FFVA between supply chain nodes was found in the level of control measures, with food service operators having less comprehensive control measures than the other groups for both technical and managerial controls. In another study on food fraud vulnerability^47^, food service operators were rated as relatively vulnerable, also due to the lower level of controls and increased opportunities and so this area of the supply chain seems to represent an area of weakness and countermeasures should be considered for the established vulnerabilities.

Business size and certification status are also investigated in this study for perceived food fraud vulnerability. However, these traits are not routinely captured in food fraud databases, so it was not possible to compare food fraud prevalence for these factors. However, a research report funded by the MSC found their certified products to have lower levels of mislabelling (less than 1%)^89^ than an average of around 30% found in a global evaluation of mislabelling^63^.

**Fig. 5:**
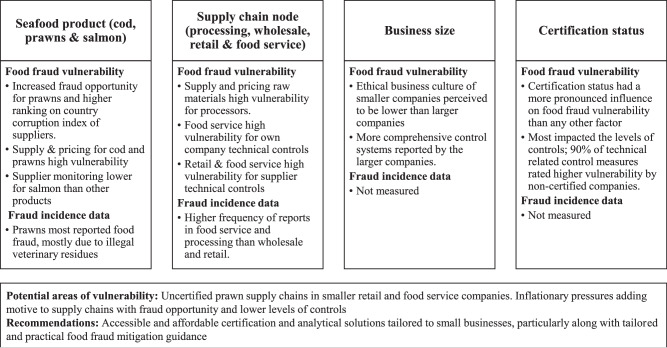
Summary of findings across commodity, supply chain node, business size and certification status. This summary includes data from both perceived food fraud vulnerability and publicly available food fraud data, to identify areas of the supply chain that could require greater scrutiny.

The overall results are presented in Fig. 5 to provide an overview of the analysis and trends observed by seafood product, supply chain node business size and certification for both perceived food fraud vulnerability, and publicly available historic food fraud data, where available. This provides the ability to identify where combined characteristics could lead to increased vulnerability, for example, uncertified prawn supply chains in small retail and food service companies.

## Discussion

This paper aimed to explore the food fraud vulnerability of UK post-harvest seafood supply chains and compare the results by commodity, supply chain node, business size and certification status. Overall, the UK’s seafood supply chains are perceived to have medium vulnerability to food fraud. Small variances were observed by commodity and supply chain node; prawns had a higher vulnerability to food fraud due to the ease of adulteration and geographic location of suppliers. Retailers and food service had a higher perceived vulnerability due to weaker own company control measures. Attaining third-party certification or using certified suppliers has the greatest influence on reducing food fraud vulnerability, as certified companies had more robust control measures than uncertified companies for over 80% of fraud factors. Given the substantial impact on vulnerability, implementing requirements for food businesses to undergo basic training or certification programs that address food fraud prevention could help reduce future vulnerability. The implementation of control measures is also affected to a smaller extent by supply chain node and company size, with food service and smaller companies more vulnerable to criminality due to less comprehensive control systems. Food fraud vulnerability data was compared with publicly available historical data of fraud in these supply chains. Prawns had the highest vulnerability and prevalence of food fraud. There were a greater number of food fraud reports in processing and food service than retail and wholesale, but the vulnerability assessment indicated retail and food service were at greater risk of food fraud due to the level of controls. This may indicate some divergence in terms of perception, particularly when concerning own company and insider threats; processors, for example, perceived the technology and knowledge to adulterate raw materials higher than final materials, even though processing techniques such as breading and glazing increase opportunity for adulteration.

Combining food fraud vulnerability data with historical trends of fraud in the sector helps to identify potential areas of current or emerging vulnerability. Areas of potential concern include uncertified prawn supply chains (due to a high prevalence of adulteration and increased fraud opportunity), salmon supply chains (due to weaker supplier controls and historical misrepresentations of weight and origin) and food service companies that had the largest number of historical fraud reports, but the least robust control systems.

Finally, many small, uncertified businesses in the UK use well-sourced, local supply chains, offering a short, sustainable route from boat to plate, but lack the resources or finances to prove or protect this provenance. Accessible and affordable certification and analytical solutions, along with simple, tailored and practical food fraud mitigation guidance could help demonstrate good traceability whilst protecting companies from disreputable operators seeking to take advantage of unsecured supply chains.

## Methods

### Recruitment and data collection

**Fig. 6:**
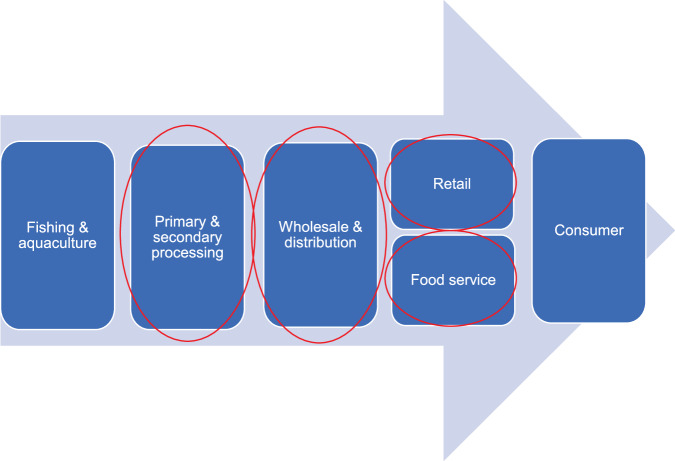
Areas of the post-harvest supply chain selected for analysis. These areas represent key stages of the supply chain process that will be used to organise the results.

Study participants for the fraud vulnerability assessments were actors in the post-harvest UK cod, prawn and salmon supply chains, including primary and secondary processors, wholesalers and distributors, retail and food service operators, but excluding consumers. Post-harvest nodes of the seafood supply chain included in the study are illustrated in Fig. 6.

Approximately 500 companies (where contact details could be located via internet searches and seafood trade association lists) in seafood processing, wholesale & distribution, retail and food service were sent details of the study via e-mail and invited to participate, followed up by a telephone call. Thirty-two companies agreed to participate and were interviewed using the SSAFE FFVA on one of three commodities, cod, prawns, or salmon. These products are included in the top 5 species consumed in the UK^64^ and represent wild, farmed, local and imported products. Where available, the interview was conducted with a representative from the company’s technical team, or by the company owner. The study was reviewed and approved by the Faculty of Medicine, Health & Life Sciences Research Ethics Committee, Queens University Belfast and written informed consent was obtained from each subject prior to their participation in the study.

The assessment was conducted face-to-face, or via video call or phone so that the researcher could clarify any questions. The assessment^37^ was sent in Excel format to these respondents ahead of the meeting, so they had time to gather necessary information and consult relevant organisational departments if required. Where companies indicated that their preference was to answer the questionnaire by e-mail, detailed information was provided by e-mail on how to fill out the questionnaire and offered telephone support. All assessments were conducted between January 2022 and April 2022 Companies interviewed were categorised by size according to UK governmental business population bandings; small (0-49 employees), medium-sized (50–249 employees) and large (250 or more employees)^65^. Location was also categorised by region, as defined by the Office for National Statistics^66^.

Also noted was any third-party certification held by the company or demanded of their suppliers (for retail and food service). There are numerous certifications applicable to the food system that enable organisations to align their supply chains with harmonised standards. For seafood, this includes certifications such as the MSC for wild-caught fish and the ASC for farmed species, fishery certification programs that indicate that seafood has been sustainably sourced in a socially responsible way. Standards certification bodies such as BRCGS, ISO and GFSI ensure companies meet international, benchmarked food safety standards.

The characteristics of survey respondents are detailed in Table 1. The distribution of participants was relatively well balanced across commodity; cod (*n* = 12), prawns (*n* = 10), salmon (*n* = 10) and supply chain node (processing (*n* = 10), wholesale (*n* = 8), retail (*n* = 8) and food service (*n* = 6)). Seventeen small companies took part, five medium and ten large. Seventeen companies had third-party accreditation, or it was included in supplier contracts.

This study uses the SSAFE FFVA assessment, a free science-based tool^37^ developed by SSAFE in collaboration with Wageningen University and Research Center and the Vrije Universiteit Amsterdam, in consultation with the food industry, regulators and intergovernmental organisations. The assessment is designed to surface food fraud vulnerabilities based on routine activity theory^67^, a key theoretical approach in criminology that focuses on situational aspects of crime. When three key elements converge in time and space: a motivated offender, a suitable target and the absence of guardianship, it is considered that crime is likely to occur.

Previous research has categorised these elements to analyse food into opportunities, motivations and controls and six further associated subcategories; technical opportunities, opportunities in time and space, economic drivers, cultural and behavioural drivers, technical controls and managerial controls^26^. The FFVA consists of 50 fraud indicators designed to surface resilience or vulnerabilities relating to these categories. Each indicator has a question that can be answered with low, medium, or high vulnerability to provide an overall picture of an individual company’s potential food fraud vulnerability. The categories and corresponding indicators are detailed in Table 4.

**Table 4:** Fraud indicators and corresponding categories

Opportunities			Motivations			Control measures		
Technical Opportunity	1	Complexity of adulteration of raw materials	Economic drivers	12	Supply and pricing raw materials	Technical measures	32	Fraud monitoring system raw materials
	2	Technology and knowledge to adulterate raw materials		13	Valuable components or attributes		33	Verification of fraud monitoring system raw materials
	3	Fraud detectability in raw materials		14	Economic health own company		34	Fraud monitoring system final products
	4	Technology and knowledge to adulterate final products		19	Financial strains supplier		35	Verification of fraud monitoring system final products
	5	Fraud detectability in final products		20	Economic health supplier		36	Information system own company
	6	Complexity of counterfeiting		26	Economic health sector		37	Tracking and tracing system own company
	7	Detectability of counterfeiting		30	Level of competition branch of industry		42	Fraud monitoring system supplier
In time and space	8	Access to production lines/processing activities		31	Price asymmetries		43	Information system supplier
	9	Transparency in the chain network	Culture & behaviour	15	Organisational strategy own company		44	Tracking and tracing system supplier
	10	Historical evidence of fraud in raw materials		16	Ethical business culture own company		50	Fraud contingency plan
	11	Historical evidence of fraud in final products		17	Criminal offences own company	Managerial measures	38	Integrity screening own employees
				18	Corruption level country own company		39	Ethical code of conduct own company
				21	Organisational strategy supplier		40	Whistle blowing own company
				22	Ethical business culture supplier		41	Contractual requirements supplier
				23	Criminal offences supplier		45	Social control chain network
				24	Victimisation of supplier		46	Fraud control industry
				25	Corruption level country supplier		47	National food policy
				27	Criminal offences customer		48	Law enforcement local chain
				28	Ethical business culture branch of industry		49	Law enforcement chain network
				29	Historical evidence branch of industry			

### Adaptation of the FFVA tool to the seafood supply chain

Questions 6 and 7 relating to counterfeiting were omitted as they were irrelevant to the businesses interviewed. In total, 48 questions were included, the numbering of the original FFVA tool was maintained to enable comparisons with other studies using the tool.

Food fraud data is held by numerous bodies, including international agencies, national enforcement agencies, local authorities and border control, where illegally traded goods are often identified. As this data is not yet collated on a single database, several resources are available to collate data on food fraud incidences. This paper uses open access data from the EU’s Rapid Alert System for Food and Feed (RASFF), HorizonScan, the Food Fraud Database and Nexis (described in Supplementary Information B, Table 4) to obtain a basic impression of historic criminality in cod, prawn and salmon supply chains.

### Data analysis

The food fraud vulnerability questionnaire provided to respondents consists of 48 questions and they were presented with three descriptions that relate to answering options for each question. These represent low, medium and high vulnerability levels for each fraud factor and are represented with a score of 1, 2 or 3. For opportunities and motivations, 1 is low vulnerability, 2 is medium vulnerability and 3 is high vulnerability. For control related fraud factors, these scores are reversed.

The frequencies of low, medium and high vulnerability scores for each fraud factor were obtained so that key fraud factors could be compared based on these scores. If the high vulnerability score was greater than 50% for any fraud factor, it was considered high vulnerability, or if the high and medium scores accounted for over 75% of the responses.

Cluster analysis was performed on the entire dataset using multiple correspondence analysis (MCA) to explore associations by commodity and supply chain node, business size and certification status. MCA condenses categorical data into a lower-dimensional space, facilitating visualisation and interpretation of patterns. A graphical output simplifies complex data structures and helps identify initial relationships and clusters among variables, which is useful for guiding further analysis. This analysis was conducted on XLSTAT (https://www.xlstat.com/en/).

To compare significant differences by commodity, supply chain node, business size or certification status, non-parametric Man-Whitney U and Kruskal Wallis tests (*p* < 0.05) were chosen as the vulnerability scores are ordinal. Using this analysis, mean ranks were compared across the groups. For comparison by supply chain node, questions 8 and 27 were omitted as they were not relevant for all supply chain nodes. This analysis was conducted using IBM SPSS Statistics software.

### Methodological considerations

Recruiting participants to take part in interview-based food fraud vulnerability studies is difficult due to the sensitive nature of the subject matter and questionnaire. It is possible that businesses who are more confident in their supply chain and sourcing practices and may already be taking proactive steps to reduce their food fraud risks are more likely to respond to an invitation to participate in such a study and so the sample may be somewhat self-selecting. However, considerable efforts were made to ensure a diverse representation of participants across commodity chain, seafood product and business size, which was successfully accomplished. Furthermore, a substantial proportion of smaller companies were incorporated (who may have less comprehensive control systems in place) and there was an even distribution between companies with and without third-party certification.

Data on historic food fraud incidences in cod, salmon and prawn supply chains is collected from publicly available databases and as much food fraud goes unreported, this data is likely to represent a small proportion of criminality in the supply chains investigated. However, the inclusion of data on reported fraud provides an opportunity to compare potential points of deception in the supply chains supply chain identified by the research to historic reports that evidence where deceptive behaviour has previously taken place. The use of publicly available databases means the data is widely accessible and the searches conducted on them are replicable.

The data were collected in 2022 and supply chains continue to be impacted by disruptions, potentially impacting the current applicability and generalisability of the findings. Future research should aim to incorporate more recent data to ensure the relevance and accuracy of the conclusions.

### Reporting summary

Further information on research design is available in the Nature Research Reporting Summary linked to this article.

### Supplementary information

Supplementary Material

Reporting summary

## Data Availability

The anonymised food fraud vulnerability datasets generated during the current study are available in the Mendeley repository. Lawrence, Sophie; Elliott, Chris; Huisman, Wim; Dean, Moira; van Ruth, Saskia (2024), ‘Food fraud vulnerability of cod, prawn and salmon supply chains’, Mendeley Data, V1, 10.17632/mfs2y6rhbj.1. The food fraud prevalence data that support the findings of this study are available from HorizonScan (https://horizon-scan.fera.co.uk/), Decernis’s Food Fraud Database (https://ffd.decernis.com/) and Nexis (https://www.lexisnexis.co.uk/). Restrictions apply to the availability of these data, which were used under license for this study. The data are, however, available from the authors upon reasonable request and with the permission of the database providers.

## Code availability

The authors declare no custom code or mathematical algorithm is deemed central to the conclusions in this study.
