# Supplementary material for: Food fraud threats in UK post-harvest seafood supply chains; an assessment of current vulnerabilities
Source: NPJ Sci Food. 2024 May 27;8:30. doi: 10.1038/s41538-024-00272-z (PMC11130318; doi:10.1038/s41538-024-00272-z)
Supplement: Supplementary file 1 — Supplementary Material [file 41538_2024_272_MOESM1_ESM.pdf]

Supplementary Table 1. Percentage of low, medium and high vulnerability responses by individual fraud factor

Question #		Low (%)	Medium (%)	High (%)
	**Fraud opportunity**			
1	Is it simple or complex to adulterate your raw materials?	63	6	31
2	Is the technology and knowledge to adulterate your raw materials generally available?	16	9	75
3	How easily can adulteration of your raw materials be detected and with what kind of methods?	25	9	66
4	How available is the technology and knowledge to enable the adulteration of your final products?	32	23	45
5	How easily would adulteration of your final products be detected and what kind of methods are available?	23	6	71
8	How would you describe the production lines / processing activities of your company?	71	19	10
	**Fraud motivation**			
9	How would you describe your part of the food supply chain?	88	6	6
10	Have fraudulent incidents of similar raw materials been reported?	65	29	6
11	Have fraudulent incidents of similar final products been reported?	63	28	9
12	How would you define the supply and pricing of your raw materials?	38	19	44
13	Do special attributes or components determine the value of your raw materials?	19	3	78
14	How would you describe the economic condition of your company?	84	13	3
15	What are the characteristics of the business strategy of your company?	84	6	9
16	How would you describe the ethical business culture of your company?	88	13	0
17	Has your company been involved in criminal offences previously?	97	0	3
18	How would you rate the corruption level (according to the Transparency International Corruption Perception Index) in the countries where your company is active?	84	6	9
19	How would you describe the financial strains imposed by your company on your direct supplier(s) ?	66	31	3
20	How would you describe the economic health of your direct supplier(s)?	65	30	4
21	What are the characteristics of the business strategy of your direct supplier(s) ?	96	0	4
22	How would you describe the ethical business culture of your direct supplier(s) ?	85	15	0
23	Has your direct supplier(s) been involved in criminal offences previously ?	60	33	7
24	Has your direct supplier(s) been a victim of food fraud committed by their suppliers, customers or other parties?	54	38	8
25	How would you rate the corruption level (according to the Transparency International Corruption Perception Index) in the countries where your direct supplier(s) and customers are active?	60	17	23
26	How would you describe the economic health across your sector of the food supply chain (i.e. your company and your direct competitors)?	34	56	9
27	Has your customer(s) been involved in criminal offences previously ?	35	65	0

28	How would you describe the ethical business culture across your sector of the food supply chain (i.e. your company and your direct competitors)?	47	34	19
29	How common are criminal offences across your sector of the food supply chain? (i.e. your company and your direct competitors)?	28	56	16
30	How would you rate the level of competition across your sector of the food supply chain (i.e. your company and your direct competitors)?	16	13	72
31	Are there price differences as a result of regulatory differences across countries?	13	33	54
	**Controls**			
32	How would you rate your company's raw material monitoring control systems' ability to detect fraud?	34	28	38
33	Are the fraud monitoring tasks of your raw material control system verified in your company?	31	28	41
34	How would you describe the fraud related parts of your final product monitoring control system of your company?	33	20	47
35	Are the fraud monitoring tasks of your final product control system verified in your company?	32	25	43
36	How extensive is the information system for internal control of mass balance flows in your company?	54	11	36
37	How extensive is the tracking & tracing system of your company?	35	35	29
38	Is integrity screening of employees common procedure in your company?	19	16	65
39	Is there an ethical code of conduct or guideline in place and embedded in your company?	56	22	22
40	Is there a whistle blowing system (system for reporting assumed fraudulent activities) in place in your company?	47	13	41
41	Do contractual requirements with your direct suppliers include elements that limit opportunities for fraud?	45	14	41
42	What best describes the fraud control system of your direct supplier(s)?	42	53	5
43	How extensive is the information system for control of mass balance flows of your direct supplier(s)?	74	5	21
44	How extensive is the traceability system of your direct supplier(s)?	58	38	4
45	How would you describe the social control and transparency of actions across your supply chain?	30	60	10
46	How well established is guidance for fraud prevention and control across your sector of the food supply chain? (i.e. your company and your direct competitors)	37	30	33
47	How would you describe your national food policy? (i.e. country-level)	59	24	17
48	How well are fraud prevention laws enforced locally?	31	59	10
49	How well are fraud related laws enforced across your international supply chain?	33	60	7
50	Does your company have fraud contingency measures in place?	33	20	47

Supplementary Table 2. Databases used to analyse food fraud prevalence

Database	Description	Search terms
RASFF Window: The Rapid Alert System for Food and Feed (RASFF) https://webgate.ec.europa.eu/rasff-window/screen/search	The RASFF window is an online database that provides notifications on risks to human and animal health. Historic data from RASFF is now limited on the portal, but available on other subscription databases such as HorizonScan and Decernis's FFD.	Bivalve molluscs and products thereof, cephalopods and products thereof, crustaceans and products thereof, dietetic foods, food supplements, fortified foods, fats and oils, feed additives, feed materials and feed premixtures, fish and fish products, gastropods, prepared dishes and snacks, soups, broths, sauces, and condiments (only where seafood is the concern).<br><br>Hazard Category: fraud/adulteration, poor/insufficient controls, veterinary residues.
Decernis Food Fraud Database (FFD) https://decernis.com/	The FFD is a searchable database of food fraud records from rom the scientific literature, media publications, regulatory reports, judicial records, and trade associations.	Incident data for ingredient group: Seafood and seafood products. This includes all fraudulent incidents for seafood recorded on the database, including illegal or unauthorized veterinary residues.
HorizonScan https://horizon-scan.fera.co.uk/	HorizonScan is owned by FERA and is a searchable database containing data on current and historical global food fraud and contamination issues	Commodity group: seafood, canned seafood products, fish oil, feed materials—fishmeal, feed materials—crustaceans, frozen ready meals, part cooked chilled ready meals, other prepared foods, soups (chilled), Soup mixes (dry), sauces, other prepared foods, and snack foods.<br>Dashboard: Fraud issues (vulnerability assessment).
Nexis https://www.lexisnexis.com/	A searchable news database from Lexis Nexis of 40,000 premium and web sources, including news and media sources, business information and legal documents	Keyword search: (seafood or *fish*) and (fraud or crime).
